# Impact of an All-Female Surgical Team on Moroccan Patient Perspectives of Female Healthcare Providers

**DOI:** 10.1007/s00268-021-06263-5

**Published:** 2021-08-07

**Authors:** Naikhoba C. O. Munabi, Meredith D. Xepoleas, Kella L. Vangsness, Sara Koualla, William P. Magee, Caroline A. Yao

**Affiliations:** 1 Division of Plastic and Reconstructive Surgery, Keck School of Medicine of the University of Southern California, 1510 San Pablo St, Suite 415, Los Angeles, CA 90033 USA; 2 Operation Smile Inc, Virginia Beach, VA USA; 3 Division of Plastic and Maxillofacial Surgery, Children’s Hospital Los Angeles, Los Angeles, CA USA; 4 Faculté de Médecine Et de Pharmacie D’Oujda, Oujda, Morocco; 5 Department of Plastic Surgery, Shriners Hospital for Children, Los Angeles, CA USA

## Abstract

**Introduction:**

Increasing numbers of women in medicine could address Morocco’s 5.5-fold deficit in surgical providers. Cultural perceptions towards women limit female advancement in healthcare. This study evaluates the impact of an all-female surgical team on Moroccan attitudes.

**Objective:**

This study aimed to evaluate how attitudes towards female healthcare professionals changed for Moroccan patients after exposure to a unique, all-female medical environment.

**Methods:**

Cleft patients were surveyed after a surgery mission with all-female volunteers in Oujda, Morocco. Analysis included quantitative, qualitative, and mixed-methods approaches.

**Results:**

Of 121 respondents (94%), 85% and 77% had prior exposure to a female nurse or doctor, respectively. 94% of respondents strongly agreed to receiving high-quality care. 75% developed increased confidence in female providers. 68% and 69% of respondents, regardless of gender (*p* = 0.950), felt that having a female nurse or doctor did not impact care. Female patients were more likely than male patients to strongly encourage female relatives to pursue medical careers (*p* = 0.027). Respondents without prior exposure to female nurses were more likely to: pursue medical careers (*p* = 0.034), believe female relatives could pursue medical careers (*p* = 0.006), and encourage them to do so (*p* = 0.011).

**Conclusions:**

Increased visibility of women improved patient attitudes towards female providers, especially in patients without prior exposure. Initiatives that increase female representation in healthcare may have greater effects in cultures with more gender inequity.

## Introduction

Eighteen million additional healthcare workers are needed worldwide to achieve universal health coverage [1]. Globally, almost one billion women do not participate in the formal workforce. As women are under-represented in physician and healthcare leadership roles, particularly in surgical fields, they are the largest demographic that can be mobilized into the medical workforce [2]. Economic and sociocultural factors discourage female participation in the workforce in certain regions. The source of this gender inequity includes cultural stereotypes, lack of encouragement and mentorship, or minimal representation of women in higher level careers [3–8].

Women in the Middle East and North Africa have 61% of the professional opportunities, status, and cultural freedoms of men, the lowest of all regions worldwide [9]. The Global Gender Gap Index (GGGI) ranks 153 countries annually according to four measures of gender equity: economic participation and opportunity, educational attainment, health and survival, and political empowerment. Morocco, a lower middle income country of 34.7 million people, ranks 143 out of 153, with sub-group rankings of 146, 115, 138, and 123, respectively [9]. The current density of surgical providers in Morocco is 3.66 per 100,000 population; they need a 5.5-fold increase in providers to achieve the Lancet Global Surgery Commission recommended 20 per 100,000 population [10]. More women in medicine could alleviate this need.

Moroccan women may be deterred from higher level jobs in medicine due to gender norms and lack of female representation in the field [11, 12]. In Morocco, women are employed one-third as often as men. Paradoxically, as Moroccan women achieve higher levels of education, their unemployment rate nearly doubles from 27.5% to 50.0% [12].

More women working in medicine changes perceptions and increases the number of women who pursue male-dominated specialties such as surgery [5]. This pilot study evaluated whether an all-female surgical team in Morocco affected patient and family attitudes towards women in medicine.

## Methods

A survey was administered during a five-day Operation Smile cleft surgery mission in Oujda, Morocco (March 2020) with an all-female volunteer team. Patients who received surgical care (cleft lip and/or palate repair) were eligible for study inclusion. After informed consent, surveys were completed on the day of discharge with an in-person translator. All surgical patients received post-operative followup care by the Operation Smile Morocco team at their regional cleft care centers after the mission according to standard organizational protocols. Patients older than 14 years were surveyed directly (“self-respondents”). Patients aged 14 years or younger had their surveys completed by a parent or guardian (“parents”). Survey questions collected the following data: patient demographics, prior exposure to female healthcare providers, and opinions about the quality of care received and perceptions about the role of women in medicine. All 12 survey questions were free response or a four-point Likert scale. A mixed-methods approach was used in this pilot study to better understand the study participants’ perspectives and provide a comprehensive overview of the findings.

Data from completed surveys were entered in REDCap (Vanderbilt University, Nashville, TN). Statistical analyses were done using Excel (Microsoft Corp, Redmond, WA) and SPSS (IBM Corp, Armonk, NY) with significance defined as *p* < 0.05. Qualitative and mixed-method analysis was performed using Dedoose (SocioCultural Research Consultants, Manhattan Beach, CA).

This study conformed to principles in the Declaration of Helsinki. Ethics approval was obtained from Children’s Hospital Los Angeles (IRB #CHLA 20–00,026) and Operation Smile, Inc. (Virginia Beach, VA).

### Patient and public involvement

Due to the environment in which this study took place, research did not incorporate patient and public involvement in study design, interpretation of results, or writing of this manuscript.

## Results

One hundred and twenty-nine patients received surgery over five days and 94% (*n* = 121) completed the survey. Average patient age was 7.7 ± 9.9 years (range 0.4 to 53 years). The majority of patients needed a parent or guardian to complete the survey due to young age (100 patients, 82.6%), and mothers (*n* = 97) were the most common respondents. Of 81 volunteers participating in the mission, 28% (*n* = 23) were doctors, 26% (*n* = 21) nurses, 21% (*n* = 17) other medical providers, and 25% (*n* = 20) non-medical providers. Table 1 details patient demographics.

**Table 1:** Demographics of survey respondents

Respondent Characteristics	N = 121
Patient age, years ± SD	7.7 ± 9.9
*Patient gender, n (%)*	
Female	53 (43.8%)
Male	68 (56.2%)
*Survey respondent, n (%)*	
Mother	100 (82.6%)
Father	3 (3.0%)
Other guardian*	7 (5.8%)
Patient (age > 14 only)†	21 (17.4%)
*Patients with prior exposure to a female healthcare worker, n (%)*	
Nurse	103 (85.1%)
Doctor	93 (76.9%)

*Other guardian includes grandmother (*n* = 3), aunt (*n* = 3), and sister (*n* = 1)

†Patient self-respondents were 42.9% female (*n* = 9)

### The perceptions of gender and quality of care received

94% of respondents (*n* = 114) strongly agreed that high-quality care was given during the mission, with no significant difference between parents and self-respondents (*p* = 0.251). The majority of people strongly agreed that quality of their care was not impacted by the gender of nurses or doctors, (69% (*n* = 83) and 68% (*n* = 82), respectively), with no difference in parents and self-respondents (*p* = 0.214 and *p* = 0.205).

**Fig. 1:**
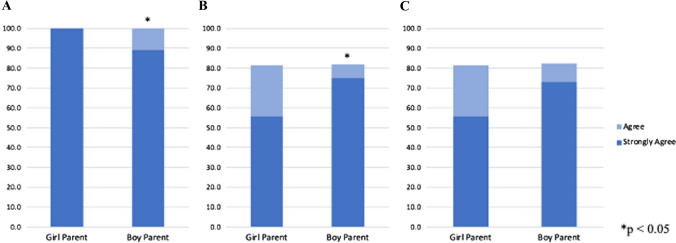
Survey responses from parents of female patients (girl parent) versus male patients (boy parent): **A** “I or my family member received high-quality care during this mission” **B** “The gender of nurses did not impact the quality of care given” or **C** “The gender of doctors did not impact the quality of care given”

Parents of female patients were significantly more likely to believe their children received high-quality care during the mission (strongly agree 97.7% vs 89.3%, *p* = 0.046) (Fig. 1A). Parents of male patients were significantly more likely to report that nurse gender did not impact the quality of care given (strongly agree, 75.0% vs 54.5%, *p* = 0.045) (Fig. 1B). Parents of boys were more likely to strongly agree that doctor gender did not impact quality of care, but this difference was non-significant (73.2% vs 54.5%, *p* = 0.071) (Fig. 1C). Respondent gender did not impact opinions of whether nurse or doctor gender affected quality of care (*p* = 0.950 and *p* = 0.950, respectively). No relationship was observed between patient age and opinion about quality of care. Impact of parent gender on survey responses was not evaluated due to the small number of male parents (*n* = 3).

### The impact of gender representation on future career aspirations

**Fig. 2:**
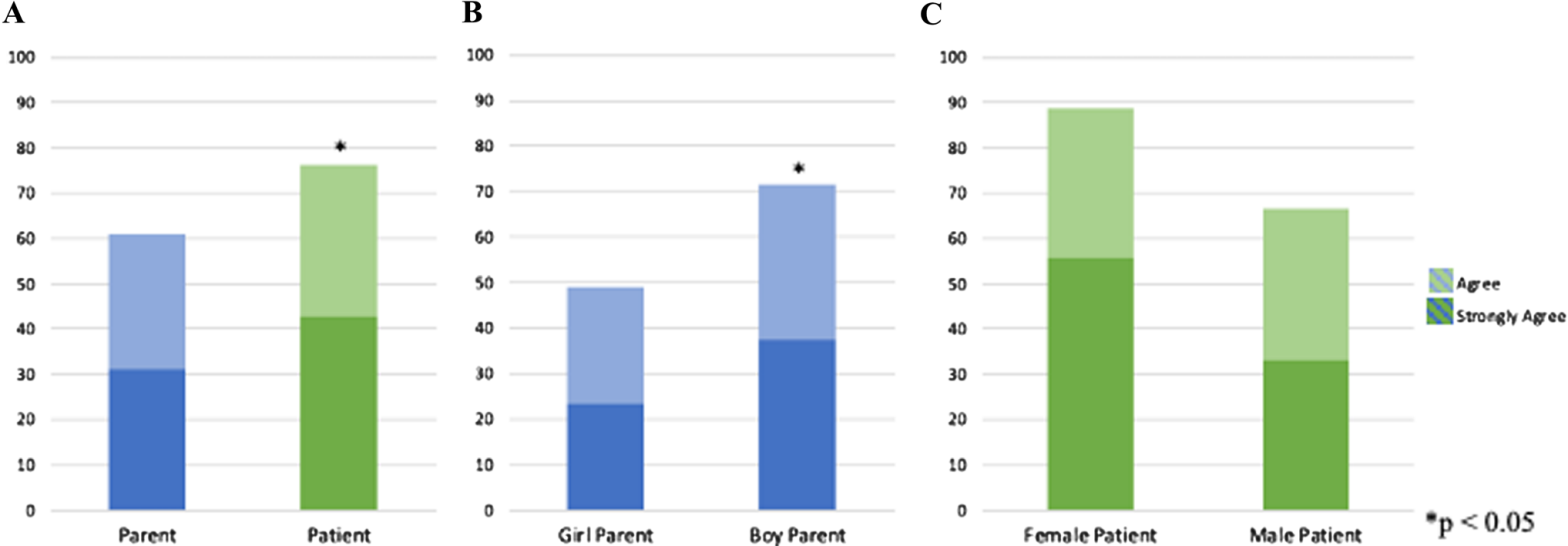
Responses to: “This experience inspired me to pursue a career in medicine or healthcare”. **A** Parent compared to patient respondents. **B** Parents of girl compared to boy patients. **C** Female compared to male patients. Responses of parents appear in blue and patients in green

75% of respondents strongly agreed that receiving care from an all-female healthcare team helped them believe they or their female family members could pursue careers in medicine. 78% strongly agreed they would encourage female family members to pursue a career in medicine. More self-respondents (43%) than parents (31%) strongly agreed they were inspired to pursue a career in medicine as a result of the experience (*p* = 0.029) (Fig. 2A).

All parents, regardless of their child’s gender, strongly agreed (74%) or agreed (26%) the mission experience made them believe in the capabilities of female family members. Of parents, 78% strongly agreed they would encourage female family members to pursue careers in medicine. Parents of boys were more inspired to pursue a career in medicine themselves as a result of the mission experience (71% vs 48%, *p* = 0.010) (Fig. 2B).

**Fig. 3:**
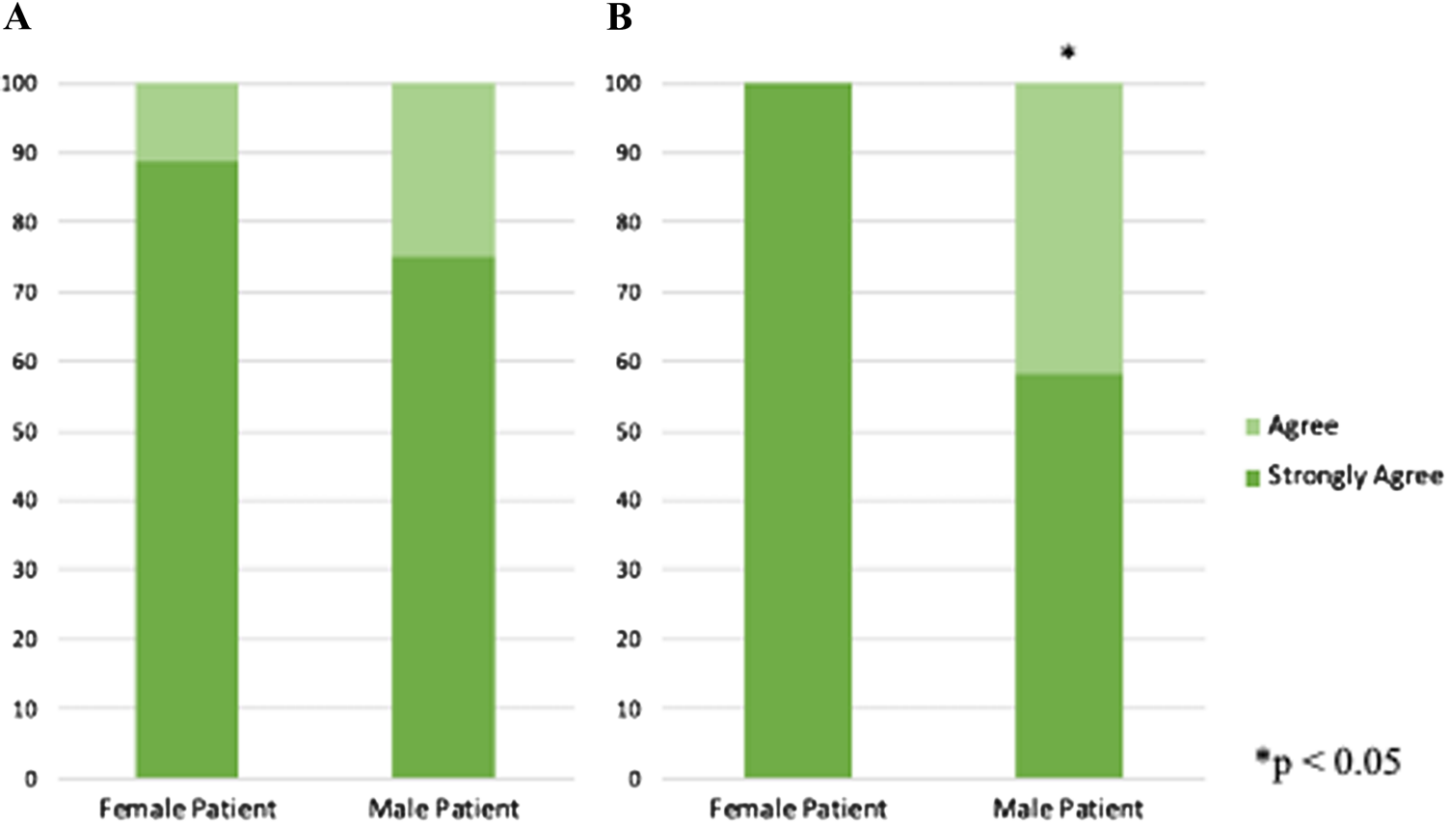
Patient self-respondent responses to: **A** “This experience made me believe my family member could work in medicine or healthcare” or **B** “Because of this experience, I will encourage my female family member to work in medicine or healthcare”

Self-respondent gender did not affect whether the mission-inspired pursuit of a medical career (55.6% female vs 33.3% male, *p* = 0.436) (Fig. 2C). After the mission experience, female and male patients both strongly agreed their female family members were capable of working in medicine (88.9% vs 75.0%, *p* = 0.422). Female patients were more likely to strongly encourage their female family members to pursue careers in medicine (100.0% vs 58.3%, *p* = 0.027) (Fig. 3). Patient age did not affect opinions about career aspirations, capabilities of female family members, or support for female family members to pursue medicine.

### Prior exposure to female healthcare workers and opinions on healthcare quality and future career aspirations

Most respondents had prior exposure to a female nurse or doctor, (85% and 77%, respectively, Table 1). Prior care from a female nurse was associated with strongly agreeing that high-quality care was received during the mission (95% vs 93%, *p* = 0.012). Respondents with previous care from a female doctor were less likely to strongly agree that high-quality care was received during the mission, though the discrepancy was minimal (95% vs 96%, *p* = 0.037). Respondents did not think female gender affected quality of care on the mission, regardless of prior exposures to female nurses or doctors.

**Fig. 4:**
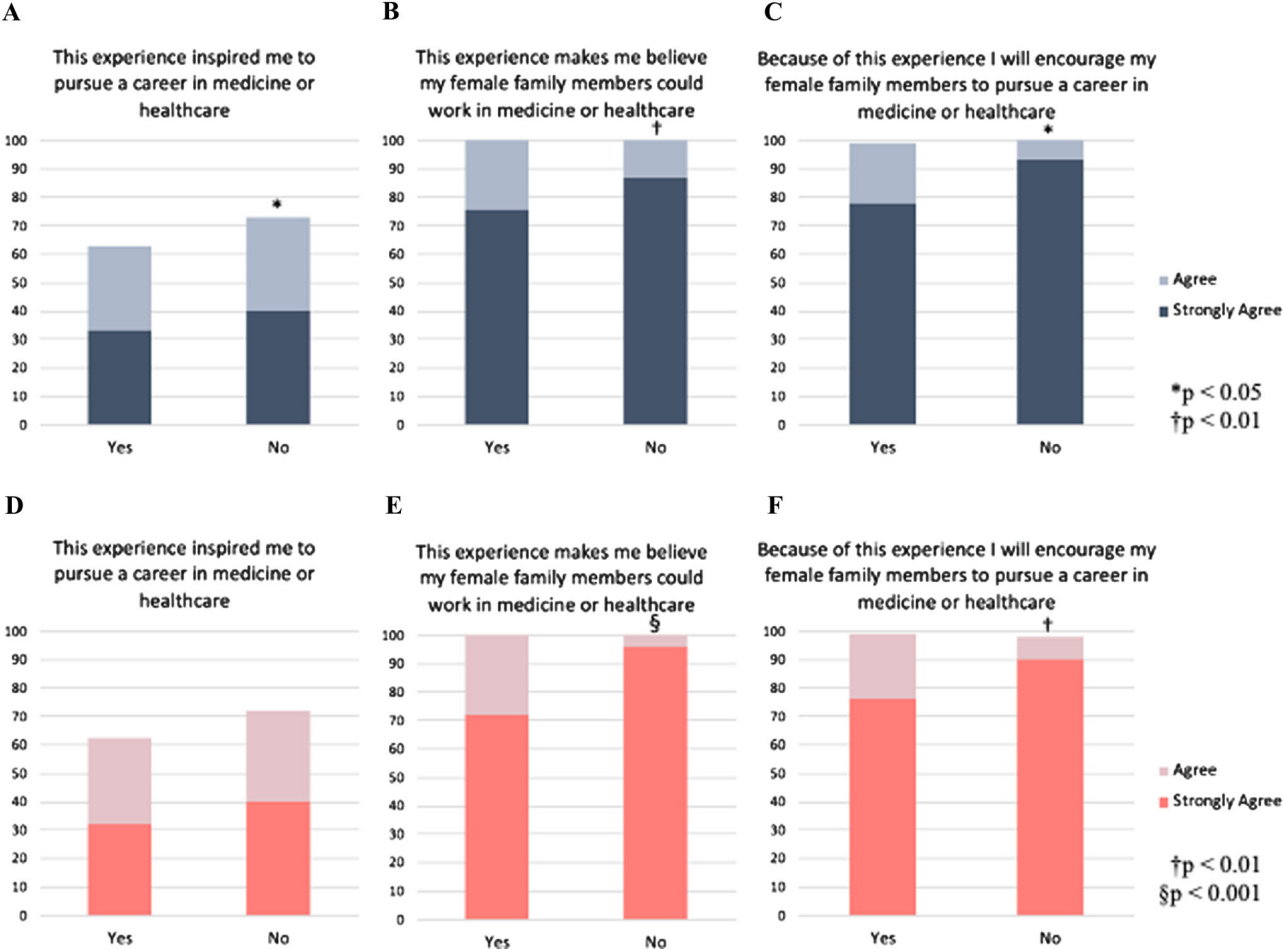
Responses of individuals who have (yes) or have not (no) had prior exposure to **A**-**C** a female nurse or **D**-**F** a female doctor. Statements included: **A** and **D** “This experience inspired me to pursue a career in medicine or healthcare”, **B** and **E** “This experience makes me believe that my female family members could work in medicine or healthcare”, or **C** and **F** “Because of this experience, I will encourage my female family members to pursue a career in medicine or healthcare”

Respondents who were interacting with a female nurse for the first time were more likely to: pursue a career in medicine (strongly agree 40.0% vs 33.3%, *p* = 0.034), believe female family members could pursue careers in medicine (strongly agree 86.7% vs 75.7%, *p* = 0.006), and encourage female family members to pursue careers in medicine (strongly agree 93.3% vs 77.7%, *p* = 0.011) (Fig. 4). Similarly, respondents who received care from a female doctor for the first time were more likely to believe female family members could pursue careers in medicine and encourage them; however, respondents themselves were not inspired to pursue a medical career (40.0% vs 32.3%, *p* = 0.579).

### Prior exposure to female healthcare providers and visibility of women in medicine

94% of respondents (*n* = 114) noticed that all healthcare providers and staff at the mission were women and 69% (*n* = 79) of them voiced surprise. No association was observed between surprise at female provider gender and respondent gender (*p* = 0.815), prior exposure to female nurses (*p* = 0.722), or prior exposure to female doctors (*p* = 0.655). Younger patients were more likely to be surprised by the all-female healthcare team (22.3 ± 7.0 years vs 31.0 ± 9.8 years, *p* = 0.027).

### Qualitative opinions on female healthcare providers

**Fig. 5:**
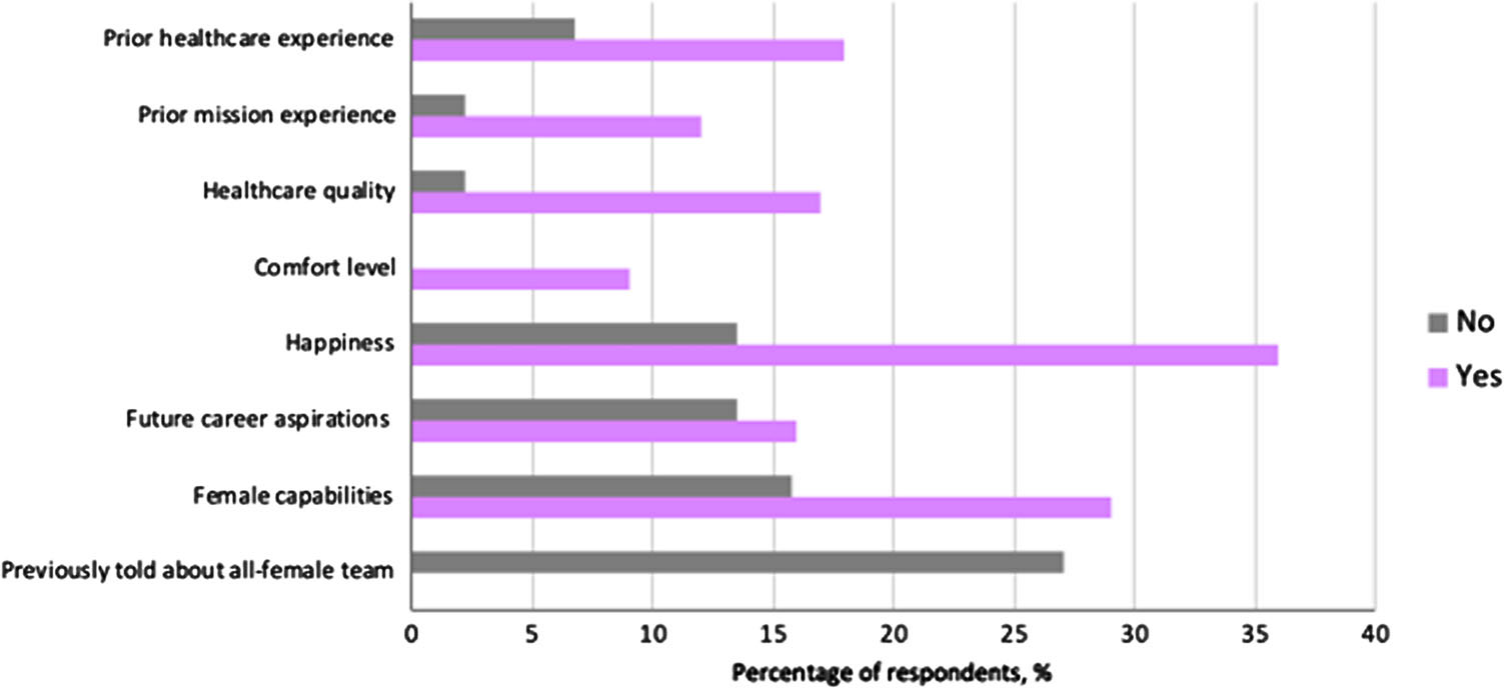
Respondent thought content after reporting whether they were surprised (yes vs no) that all of the volunteers were women

The most common thoughts raised by respondents included amazement regarding female capabilities and happiness from observing women in medical roles (Fig. 5). Many were surprised to see women in a surgeon’s role, which was previously perceived as a man’s role.

“I thought that surgeons [were] supposed to be men only.”—*Mother of a male patient.*

“I was waiting for a man, but no man came to visit me.”—*Aunt of male patient.*

Many reported the perception that women are not capable of performing surgery:

“[I] always hear that women can do nothing without men.”—*Mother of a male patient.*

“[I] was scared—[I] thought maybe the surgery won’t work because they are women. But I regret that now.”—*Mother of a female patient*

Many respondents, particularly mothers and older female patients, were pleasantly surprised to see capable female healthcare workers. They felt more comfortable with female volunteers, which led to more candour.

“I liked this mission because there are only women and they can understand [each] other more than men.”—*Female patient*

“I can ask anything to a woman and could not be the same if there's a man.”—*Mother of a female patient*

“I'm wearing a Niqab so I can take it off and be myself because there's only women.”—Mother of a female patient.

Thirty-five respondents (31%) who noticed that the team was all women were not surprised by this gender distribution.

“Where I live there’s only a woman doctor and woman nurse, so I felt comfortable.”—Mother of a female patient.

“I believe in equality and women are like men. They can do everything alone.”—*Male patient*

## Discussion

Despite multiple initiatives to increase women’s economic participation in the past two decades, Morocco has struggled improve gender equity and has a low GGGI ranking [9, 13–15]. In the Moroccan healthcare sector, this gender divide is reflected in women comprising 59% of medical students but only 25% of teaching staff and 30% of academic research positions [16–18]. Women working in medicine is commonplace in Morocco, as the vast majority of study participants had previously received care from a female nurse (85%) or doctor (77%). Despite this exposure to female healthcare workers, qualitative analysis revealed a persistent perception that medical roles, particularly those of physicians and surgeons, should be filled by men. Those who had previous interactions with a female doctor were less confident in getting good care on the mission.

Women as nurses invoked more positive responses from patients and parents than women as doctors. This may stem from nurses spending more time with families, more societal familiarity with women as nurses, and nursing seen as a more traditional female role. A 2018 study of an intensive care unit in the USA found that nurses spend 33% of their time at the patient bedside compared to 15% for physicians [19]. With more nurses than physicians in the patient wards and longer nursing interactions with patients, female nurses may generate more inspiration in patients, particularly if they were already familiar with a woman in that role. The majority of respondents were surprised by the all-female team, particularly the physicians. Patients expressed being fearful of the quality of care they would receive because of the all-female team. After the mission, patients were impressed with their care, which emphasizes that the quality and skill of a medical provider is not dictated by gender. More time and interaction with female doctors may help to reinforce this perspective and is needed to inspire future careers in male-dominated roles. Female physicians having less ability to positively influence patients and families may be a manifestation of the societal perceptions that prevent women from achieving higher level professions and leadership positions. While older respondents were hypothesized to be less accepting of women in medicine; our data showed no relationship between age and positive perceptions.

Our respondents’ high comfort level conversing with female providers is reflected in prior studies in the Middle East and North Africa. In a patient satisfaction study of 375 patients seeking pain management services in Jordan, 56% of women preferred female doctors, whereas 45% of men preferred male doctors [20]. A 2016 Dutch study investigated health checks for Moroccan and Turkish immigrants and found that women preferred gender-matched healthcare providers [21]. The gender of providers may influence patient and family comfort in cultures with more traditional gender norms. The ease of a female-to-female interaction may account for this study’s 94% participation rate, as 81% of respondents were female. The high prevalence of female respondents may bias our findings towards predominantly positive sentiments and towards women in medicine.

Female patients and their caretakers felt more vulnerable regarding the gender of their healthcare provider, as parents of girls were more likely to perceive an association between medical staff gender and quality of care received. A related USA study found that 63% of girls compared to 27% of boys had a gender preference for their paediatrician. Over 97% of girls preferred a female doctor compared to 53% of boys requesting a male doctor [22]. Parents may sense their child’s increased comfort with a gender-matched healthcare provider, explaining why parents of female children in our study expressed higher satisfaction in an all-female environment.

The all-female team improved perceptions of women in medicine for nearly all respondents, regardless of gender. Male and female patients were equally likely to believe in the capabilities of their female family members after the mission, but women were more likely to act on this sentiment. Personal inspiration to join the medical workforce occurred less often in older parents who were less likely to have resources, time, or support to change or enter a new career. To increase the number of women in medicine, a difference in attitude towards female providers coupled with personal inspiration is necessary to change gender representation. Our findings suggest that female caretakers and female patients who see women in medicine generate the strongest influence on future generations.

The impact of female representation in medicine may be greater in communities more naïve to the concept of women working in medicine. After the all-female mission, patients who received care from a female nurse for the first time were more likely to believe in the capabilities of women in medicine, encourage female family members to enter medicine, and feel personally inspired to participate in the medical workforce. The profound effect of female representation in medicine on future generations may be harnessed in environments with the most gender inequity, such as Morocco and other North African and the Middle Eastern countries [9].

### Limitations

Long-term impacts are unknown, as patients in our study only anticipated their future behaviour towards female family members or their personal career trajectory. Given the vulnerable population, the study did not have a pre-intervention survey to prevent respondent concern that access to healthcare might be contingent on study participation. Another limitation includes the small number of male respondents, particularly among parents. Studies with more men are needed to understand whether they are affected differently by observing women in medicine. Also, the majority of respondents were parents of younger children, who may have different perceptions compared to patient self-respondents. Lastly, the descriptive nature of this study and the small sample size limit its ability to determine significance, but as a pilot study evaluating a unique topic in the literature, these findings still remain important to disseminate.

## Conclusions

Placing women in visible positions in medicine can alter attitudes towards female medical providers and influence patients to pursue medical careers themselves. Women in more familiar roles such as nursing may generate more comfortable and positive responses at the first, when women as physicians are sparse. Nonetheless, more female physicians and medical leaders are necessary to create a visible group that may inspire future generations of women. Positive impacts of female representation are more striking in cultures with substantial gender inequity. As many countries struggle to provide enough healthcare workers for their population, initiatives that promote gender equity in medicine could bridge the gap through engaging women.
